# Craniofacial Growth and Asymmetry in Newborns: A Longitudinal 3D Assessment

**DOI:** 10.3390/ijerph191912133

**Published:** 2022-09-25

**Authors:** Ai-Lun Lo, Rami R. Hallac, Shih-Heng Chen, Kai-Hsiang Hsu, Sheng-Wei Wang, Chih-Hao Chen, Rei-Yin Lien, Lun-Jou Lo, Pang-Yun Chou

**Affiliations:** 1 Department of Plastic and Reconstructive Surgery, Chang Gung Memorial Hospital, Taoyuan 33302, Taiwan; 2 Craniofacial Research Center, Chang Gung Memorial Hospital, Taoyuan 33302, Taiwan; 3 Analytical Imaging and Modeling Center, Children’s Health Dallas, UTSW Medical Center, Dallas, TX 75390, USA; 4 Division of Neonatology, Department of Pediatrics, Chang Gung Memorial Hospital, Linkou 33302, Taiwan; 5 Department of Biomedical Engineering, National Yang-Ming University, Taipei 11221, Taiwan

**Keywords:** longitudinal assessment, 3D image, newborns, craniofacial morphology, head volume, growth chart, color-asymmetry maps, craniofacial growth

## Abstract

Objective: To evaluate the development of the craniofacial region in healthy infants and analyze the asymmetry pattern in the first year of life. Methods: The participants were grouped by sex and age (1, 2, 4, 6, 9, and 12 months) to receive three-dimensional (3D) photographs. Stereoscopic craniofacial photos were captured and transformed into a series of craniofacial meshes in each group. The growth patterns of the anthropometric indices and the degree of craniofacial asymmetry were measured, and average craniofacial meshes and color-asymmetry maps with craniofacial asymmetry scores were calculated. Results: A total of 373 photographs from 66 infants were obtained. In both genders, the highest and lowest growth rates for all anthropometric indices were noted between 1 and 2 months and between 9 and 12 months, respectively. Overall, male infants had higher anthropometric indices, head volume, and head circumference than female infants. The craniofacial asymmetry score was presented with a descending pattern from 1 to 12 months of age in both sex groups. Both sex groups showed decreased left-sided laterality in the temporal-parietal-occipital region between 1 and 4 months of age and increased right frontal-temporal prominence between 6 and 12 months of age. Conclusions: A longitudinal evaluation of the craniofacial growth of healthy infants during their first year of life was presented.

## 1. Background

The human craniofacial structure rapidly changes during the first year of life, and the amount of head growth differs with age and sex [1]. However, limited data regarding craniofacial development in healthy infants during the first year of life are available, especially for Asian infants. Establishing a standard reference of three-dimensional (3D) craniofacial models in healthy infants and assessing the natural course of head asymmetric growth pattern is critical and imperative to evaluate the severity of craniofacial anomalies in pediatric patients, plan treatments, and evaluate outcomes [2].

Traditionally, two-dimensional (2D) cephalometry has been commonly used to evaluate craniofacial form. However, 2D measurements cannot assess the detailed surface morphology as that in the 3D form, and transferring 2D cephalometry to 3D craniofacial structures can be challenging [3]. Furthermore, evaluating a complex 3D face in two dimensions does not allow facial measurements with a dimensional depth of anatomical points [4]. It is generally difficult to perform a three-dimensional CT or MRI scan in young infants because it involves radiation exposure and often requires sedation treatment [5]. In contrast, three-dimensional stereophotogrammetry is a non-ionizing and rapid imaging tool that is becoming part of the routine evaluation of patients with facial differences [6]. Sophisticated mathematical tools have been developed to quantitatively assess the 3D craniofacial models, establish the normative craniofacial volumes, and calculate the degrees of craniofacial asymmetry.

We prospectively and longitudinally assessed the development of craniofacial morphology in the first year of life and quantified the growth patterns of craniofacial height, depth, width, and volume; body height and weight; body mass index (BMI); and head circumference in Taiwanese infants. In addition, we developed time series composite 3D models during the first year of life to assess craniofacial asymmetry and growth and provide a better understanding of the normal morphologic craniofacial development.

## 2. Materials and Methods

### 2.1. Participants

Infants whose parents were both classified as having Asian ethnicity were eligible to participate in our study. A series of 3D stereophotogrammetry scans were obtained to study craniofacial growth during the first year of life. We analyzed 3D photographs of female and male infants with normal craniofacial conditions. The exclusion criteria were as follows: growth curve <3% or premature, congenital craniofacial anomaly, hormonal, chromosomal, thyroid, and bone anomaly.

### 2.2. Acquisition of 3D Images

**Figure 1:**
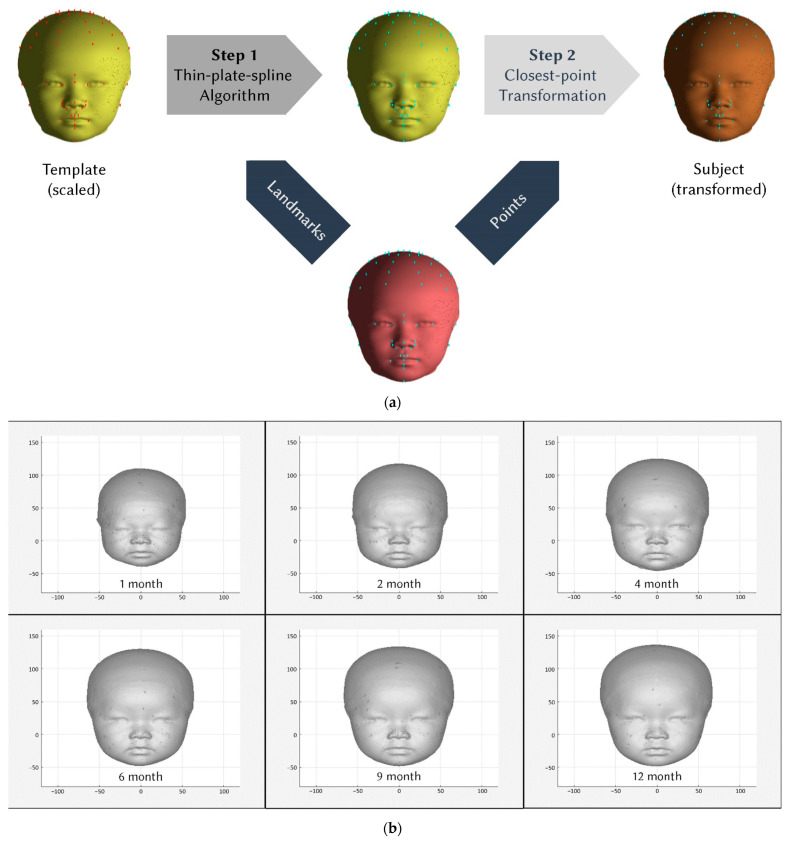

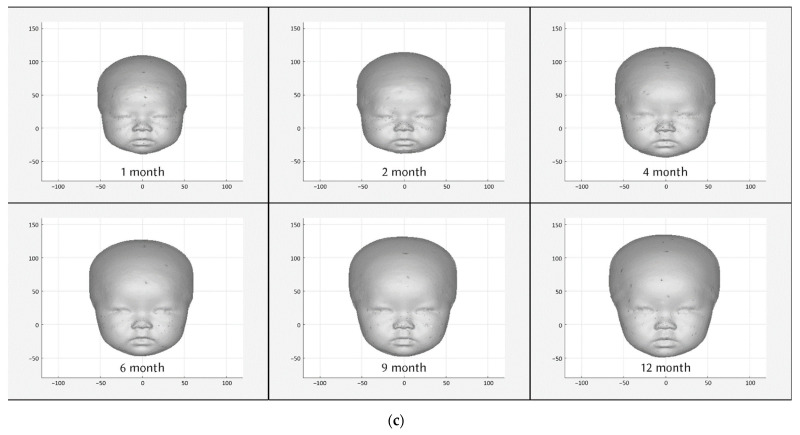
Transformation of the templates to present the average craniofacial meshes of representative newborns in the age of each month. (**a**) The figure presents the method of 3D template remodeling to the target subject. The thin-plate spline algorithm deforms the scaled template based on corresponding landmarks on the subject. Then, the closest-point deformation further details the deformed template to precisely present the subject. (**b**) Average craniofacial meshes of female infants at 1, 2, 4, 6, 9, and 12 months. (**c**) Average craniofacial meshes of male infants at 1, 2, 4, 6, 9, and 12 months.

A series of 3D craniofacial photos were captured using a 3dMD Head System (3dMD, Atlanta, GA, USA) within 7 days before or after the age of 1, 2, 4, 6, 9, and 12 months (Supplementary Materials Figure S1, Figure 1). The distance between the infants and the 3D system was 1 m, and the 3D system was calibrated weekly over time. During image acquisition, the participants were seated in a booth, and a thin elastic nylon wig cap was placed on their heads to obtain accurate head shape scans. A series of stereoscopic images were captured several times at each visit, and every effort was made to obtain accurate images with neutral facial expressions.

### 2.3. Selection of Qualified 3D Images

A series of 3D images of the participants at 1, 2, 4, 6, 9, and 12 months of age were selected if they met the following inclusion criteria: (1) a neutral facial expression with a natural head position; (2) no obvious mesh deformities that are due to uncaptured 3D images in the facial region between the ears and between the hairline and the menton.

### 2.4. Head and Body Measurements

Head circumference was manually measured using a tape. The tape was placed above the supraorbital ridges at the front, above the ears, and right across to the maximum occipital prominence at the back of the head. In addition, body height, body weight, and BMI were collected at each visit.

### 2.5. Whole-Head 3D Image Processing

A perfectly symmetric model with known left- and right-point correspondences was created using custom programs written in MATLAB (MATLAB R2015b, MathWorks, Inc., Natick, MA, USA) [7]. The facial area was defined as points with less Euclidean distance to the nasal tip than between the nasal tip and gnathion [8]. Thirty-two landmarks of recognizable anatomical structures were manually selected and placed on each 3D image using 3dMD Vultus. To make the template fit our model as closely as possible, forty additional landmarks were constructed digitally and dispersed into four layers surrounding the skull apex, which is the point where the *y*-axis (vertical dimension) meets the skull. The layers were defined at 10° decrements from the apex, with each layer comprising 10 evenly distributed landmarks. The origin of the coordinate system for each participant’s 3D model was calculated using the nearest projection of the nasal tip to the line connecting the bilateral tragus. Several steps were performed to transform the template into each participant’s 3D surface scan. First, the template was scaled to the participant’s size. The participants’ scans were then registered to match the template through rigid translation. A thin-plate spline algorithm was then applied to transform the template to each 3D scan based on the relationship between the two sets of landmarks. Finally, closest-point deformation was performed to confirm the details of the points (Figure 1a). These methods were similar to those described in our previous study [8]. The Euclidean distances from the origin of the coordinates to each point on the 3D scan were calculated.

### 2.6. Anthropometric Measurements from 3D Scans

Head height (distance between the vertex and the gnathion); head depth (distance between the glabella and the opisthocranion); and head width (distance between the euryons) were calculated following Farkas’ anthropometric definitions [9]. The head volume was calculated from the transformed template of each patient. The transformed template was divided through the sagittal midplane. Two separate templates were further calculated by establishing a 400 × 400 grid with a setting area of 1 mm^2^ on the Y-Z plane and projected on the Y-Z plane. The head area was selected above a cutting line passed through the submental and spinal processes of the C1 bone. The edges of the templates were marked with multiple black dots, and the X-coordinate values of the dots represented the height of the corresponding grids. The X-coordinate values of the grids were calculated using the average value of the black dots. Through processing, each grid had its corresponding height; thus, the total volume of each grid would be half the template volume. The sum of the left and right half templates was the total volume of the head. The Euclidean distance from the center of the head (the midpoint of the left and right tragus points) and every point on the transformed template was calculated, which was used to calculate the craniofacial asymmetry for each point and subject [10]. Color-asymmetry maps were created, which represented the mean craniofacial asymmetric pattern in each sex at 1, 2, 4, 6, 9, and 12 months of age.

### 2.7. Statistical Analysis

Quantitative variables are presented as the mean and standard deviation. Twelve average craniofacial mesh subgroups were obtained after stratifying by sex and age (male and female groups at 1, 2, 4, 6, 9, and 12 months of age). A Student’s independent-samples *t*-test was used to compare the mean values of anthropometric indices between subgroups. Scatter plots were used to plot the relationship between head circumference and other craniofacial norms, and the coefficient of determination (R^2^) was calculated. The male and female percentile curves of head volume and head circumference at selected months were shown as a normative average. SPSS version 21.0 (SPSS Software, Chicago, IL, USA) was used for the analysis, and *p* ≤ 0.05 was adopted as the level of significance.

## 3. Results

A total of 66 participants (33 female and 33 male infants) met the inclusion criteria. Three-dimensional photos were obtained for each patient at 1, 2, 4, 6, 9, and 12 months of age, leading to 373 images of female and male infants, respectively. These photographs were grouped by age and sex, and 3D composite craniofacial meshes were calculated (Figure 1b,c; Supplementary Materials Figure S2a,b). The mean head height, depth, width, volume, head circumference, body height, body weight, and BMI (Table 1) were slightly larger in male infants than in female infants. The following parameters exhibited significant between-sex differences: head height at 2, 4, 6, 9 and 12 months of age; head width at 2, 4, 6 and 9 months of age; head volume at 2, 4, 6, 9 and 12 months of age; body height at 2, 4 and 6 months of age; body weight at 2, 4, 6 and 9 months of age; head circumference at 4, 6 and 9 months of age; and BMI at 2, 4, 6 and 9 months of age. In contrast, head depth was not significantly different between sexes.

The average growth rate per month of craniofacial norms in both sexes was measured. In both genders, the highest growth rates for head height, depth, width, volume, body height, weight, and head circumference were noted between 1 and 2 months, and the lowest growth rates were noted between 9 and 12 months. In male infants, the smallest growth rate in head width was observed between 9 and 12 months. In contrast, in both genders, the largest growth rates for body mass index (BMI) were noted between 1 and 2 months and reached the maximal value at 4 months of age in female groups and 6 months of age in male groups.

**Figure 2:**
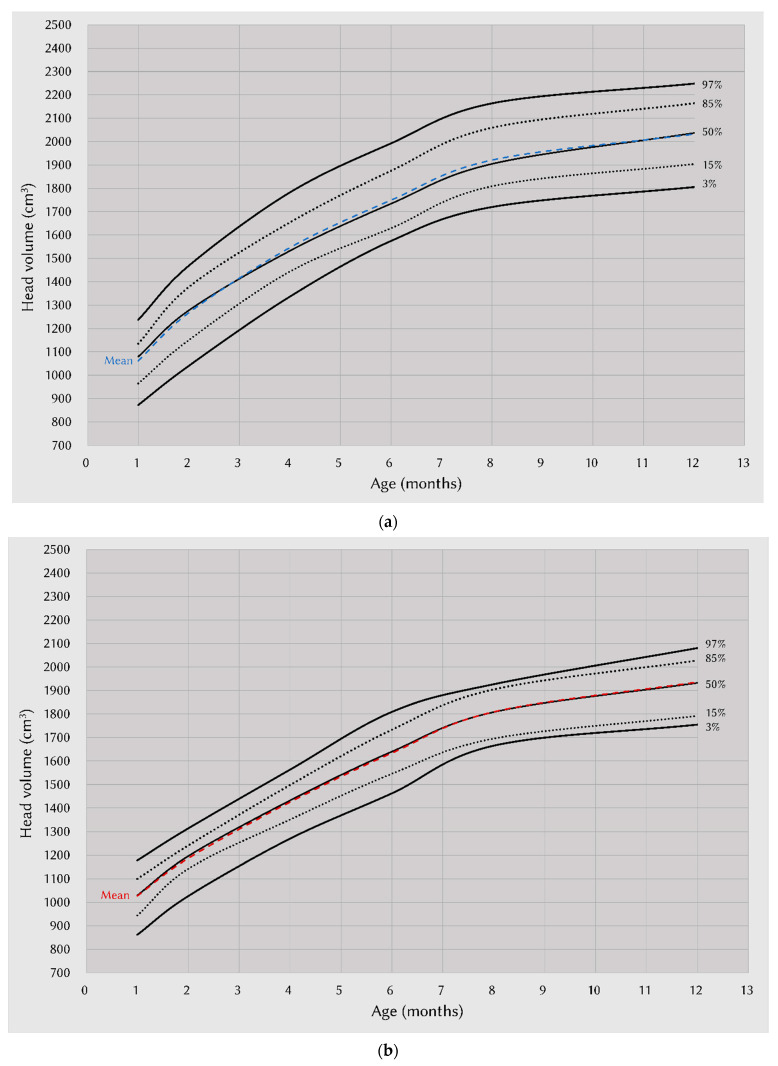
Age-specific percentile curves for head volume, stratified by sex. Percentiles of head volume per age group in males (**a**) and females (**b**). (**a**) Head volume-for-age chart: male infants, one month to one year of age. (**b**) Head volume-for-age chart: female infants, one month to one year of age.

**Figure 3:**
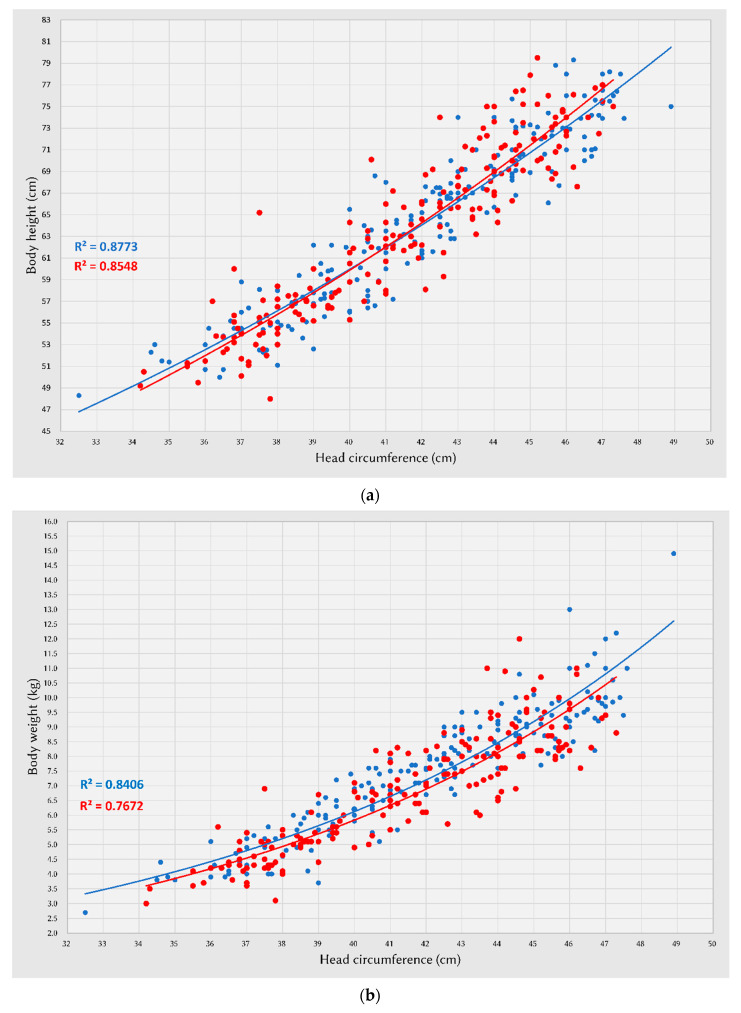

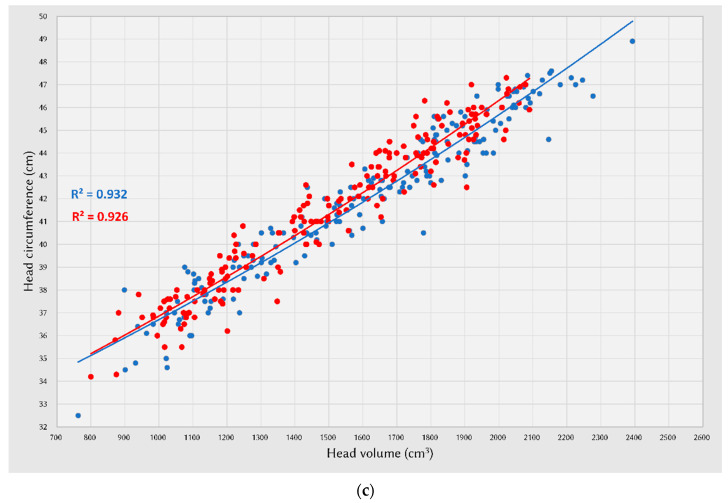
Scatter plots of head circumference with body height, body weight, and head volume in males (blue) and females (red), respectively. Regression lines are shown for the two groups separately. (**a**) Body height-to-head circumference scatter plot: male and female groups (R^2^ = 0.8773 in males, 0.8548 in females). (**b**) Body weight-to-head circumference scatter plot: male and female groups (R^2^ = 0.8406 in males, 0.76722 in females). (**c**) Head circumference-to-head volume scatter plot: male and female groups (R^2^ = 0.932 in males, 0.926 in females).

The data (3rd, 15th, 50th, 85th, and 97th percentiles and mean values for head volume) were extracted into spreadsheets. The male and female percentile curves from each included data set for head volume and head circumference were plotted so they could be visualized for their heterogeneity. The combined data were represented by fitted curves (Figure 2a,b). We compared the association between head circumference and body weight, body height, and head volume parameters between different sexes. Scatter plots demonstrated a highly positive correlation between head circumference and body weight, body height, and head volume in both sexes, especially between head circumference and head volume (R^2^ = 0.932 in males, 0.926 in females) (Figure 3a–c).

The mean craniofacial asymmetry score in males was 3.21, 2.90, 2.60, 2.52, 2.47, and 2.13 mm, and the score in females was 2.87, 2.71, 2.31, 2.16, 1.91, and 1.88 mm at the ages of 1, 2, 4, 6, 9, and 12 months, respectively (Table 1). A decreasing tendency of the mean craniofacial asymmetry score in both genders was shown. No statistical difference (*p* < 0.05) was observed in both sexes.

**Figure 4:**
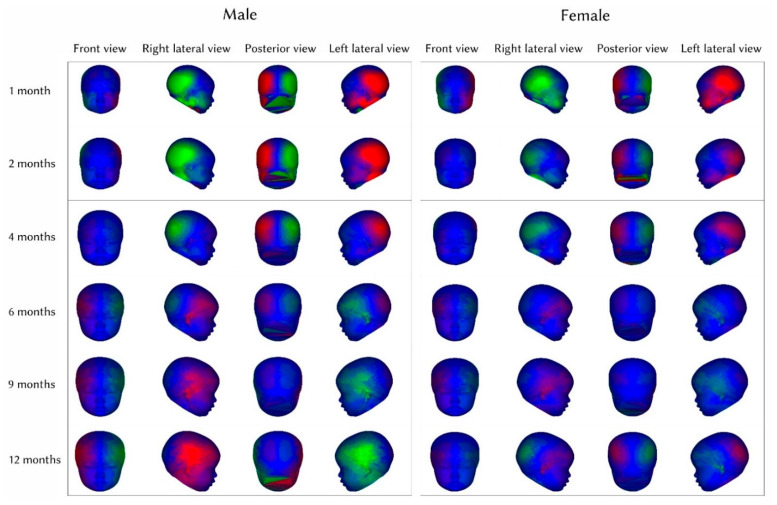
Front, bilateral and posterior view of color-asymmetry maps at 1, 2, 4, 6, 9, and 12 months of age in male (**left**) and female groups (**right**). Colors with corresponding values represent the different extent of asymmetry on this model. Green is relatively concave, and red is relatively prominent to the contralateral point.

The color-asymmetry maps of male and female infants showed unified left-sided laterality in the temporal-parietal-occipital region, and the left hemisphere prominence decreased with age between 1 month and 4 months. From 6 months to 12 months of age, the female infant groups showed increased right frontal-temporal prominence with age; in male groups, increased right frontal-temporal prominence with age was presented, while left-sided prominence in the temporal-parietal-occipital region decreased after 6 months of age (Figure 4).

## 4. Discussion

The rapid growth of craniofacial morphology in infants is ideal for longitudinal studies, especially in the first year of life. Based on our experiences with evolution [8,10], we adopted the research methods and 3D image processing in this study to assess the craniofacial morphology of healthy Asian infants aged less than 1 year-old.

### 4.1. Data Interpretation of Craniofacial Norms

The growth status of infants is critical for both parents and pediatricians. Under normal nutritional and health conditions, body height, weight, and head circumference are significantly related, and the general proportionality is reflected in all ages [11]. Accordingly, we used height, weight, head circumference, and other parameters to monitor the growth of infants. In our study, the overall growth rate of head height, depth, width, volume, and circumference, body height, and weight slowed down in both sexes as they aged, with the highest growth observed between 1 and 2 months of age and the slowest between 9 and 12 months. However, head width in 9 to 12 months showed a negative growth rate, and this can be attributed to the slower growth of head width relative to other parameters, which may be affected by the orofacial movements of the infants, such as pouting, crying, pursing the lips, or the movement of the tongue causing errors in the craniofacial measurement, even though the head volume is progressively enlarged. The growth rate of BMI tended to increase after birth; however, at 4 months of age in females and 6 months of age in males, the BMI growth rates reached their peak values and started to decline thereafter. Alherbish et al. demonstrated that the BMI value peaked at approximately 9 months in a study involving children under 2 years of age [12]. Roy et al. presented BMI charts for 73,949 full-term infants in the Pediatric Care Network Study, indicating that BMI values peaked between 6 and 9 months of age [13]. The 2014 Danish study analyzing height, weight, and BMI for participants from birth to 20 years concluded that BMI peaked at age 8.4 and 7.7 months in female and male groups, respectively [14]. These results indicate that the BMI growth rate peaked at approximately 6 to 9 months of age, which may affect the body weight and height growth rates. Although the WHO suggested that the BMI chart is not recommended for clinical use compared with weight-for-length measurement, recent studies have supported the use of BMI for growth and nutritional status assessment in infancy [15]. Moreover, our study provides solid evidence to suggest that body weight/height-to-head circumference scatter plots are highly associated.

### 4.2. Growth Charts of Head Volume and Head Circumference

To assess the development of infants during clinical visits, physicians often rely on standard growth charts. In 2006, the WHO published international growth standards for children younger than 5 years, and growth reference charts of head circumference in the United States were also produced [16]. Other studies have proposed anthropometric standards for the postpartum growth period of fetuses, newborns, and premature infants, which were modified and expanded the age range of growth standards [17,18]. Among all the parameters, head circumference represents one of the most predictable values of infant development [19,20]. Growth charts of head circumference and head volume were presented because of the high correlation of head circumference with head volume. This suggests that head volume can be used as a reference standard for growth instead of head circumference [21]. Furthermore, head shapes vary considerably among infants [22], and the presence of nonsynostotic plagiocephaly cannot be ignored [23]. Thus, using fast, convenient, and accurate instruments for craniofacial morphologic analysis to obtain head volumes may provide better results than conventional tools.

### 4.3. Interpretations of Craniofacial Asymmetry Score and Color-Asymmetry Maps

The maximal craniofacial asymmetry score was in the first month of life in both genders, and the minimal score was at the age of 12 months in both groups. We noticed that there was a tendency of decrease and no sexual difference in the craniofacial asymmetry scores. The measurements are unusual if the infant has cephalohematoma or caput succedaneum [24], leading to a disproportional growth rate of head growth in 1 to 2 months of age. The color-asymmetry maps of female and male infants from 1 month to 4 months show left-sided temporal-parietal-occipital prominence, and this result may be highly attributed to the “back to sleep” campaign, which is one of the major causes of plagiocephaly [25,26,27]. However, the temporal-parietal-occipital prominence subsided in the transitional period of 4 months to 6 months of age when all the infants were supposed to rotate their neck freely to naturally symmetrize the parietal-occipital region. From 6 months to 12 months of age, with the diminishing asymmetry over the posterior skull, the right-sided laterality in the frontal-temporal-parietal region became noticeable, and the result was similar to a previous study [8]. These results allow us to form a hypothesis that the instability of head shape can be seen under the age of 6 months. Due to notable gaps in cranial sutures and fontanelle before 6 months of age in infants, elastic space for cranial growth was preserved, and the head shape can easily be affected by head position. When some of the cranial sutures and posterior fontanelle are closed, the smaller the space is that remains at the anterior fontanel in infants aged around 6 months [28,29], and the head shape becomes relatively stable thereafter. Thus, the dramatic prominence changes between the age of 4 and 6 months in the color-asymmetry maps were seen. However, the specific mechanisms to form right-sided laterality in males are still not understood and may be worthy of further observation.

### 4.4. Strengths and Limitations of 3D Stereophotogrammetry

With the advancements in technology, many novel measurement tools have been developed. Mendonca et al. compared cranial anthropometric measurements by traditional calipers with CT scans and 3D photogrammetry [30]. The results revealed that measurements based on CT scans and 3D photographs were more reliable and interchangeable than traditional caliper measurements, which may underestimate anteroposterior and biparietal values compared with digital imaging. Due to the risk of radiation exposure and the cost of CT scans, 3D surface imaging systems have gained popularity in clinical applications. In addition to the shorter scanning time, they can also reduce errors caused by manual scanning. The ability of imaging systems to capture 3D images with both shape and texture information has made them useful for surgeons performing preoperative simulation and surgical planning [31,32]. Three-dimensional stereophotogrammetry may be reliable and precise for studying craniofacial forms [33]. However, this technology has some disadvantages: poor ear coverage [34], patient motion, or hair can introduce measurement errors. Furthermore, because of its cost, size and complexity, this system is often not suitable for clinical settings where resources, space, and time are limited [35]. In our study, we used a wig cap to obtain a better estimation of the scalp and to reduce gaps in the 3D images, but hair distribution and wrinkles from the wig cap itself could cause secondary drawbacks. Although efforts were made to acquire the images with a neutral facial expression, some infants were not fully compliant, and slight facial expressions may have created bias in our measured parameters. Furthermore, gene mutations also have certain influences on the formation of craniofacial asymmetry, which was not considered and excluded from the study [36,37]. Nevertheless, these drawbacks could be improved by properly adjusting the measuring modality, collecting more participants, using a better fitting cap, and minimizing facial expressions during the scans.

### 4.5. Application of Study Results

Our research may provide future applications in various fields, such as craniofacial deformities, low-birth-weight infants, psychiatry, and neurodevelopmental problems. Using better anthropometric tools can be helpful to evaluate the risks of primary CNS tumors [38], monitor infant microcephaly [39], identify low-birth-weight infants [40], measure the severity of deformational plagiocephaly [41], or utilize artificial intelligence in deep learning for the early diagnosis and treatment of craniosynostosis [42]. Deformational plagiocephaly (DP) is a common condition in infants that can cause craniofacial abnormalities [25], which has become more common since the 1990s “Back-to-Sleep” initiative to prevent sudden infant death syndrome (SIDS) [43,44,45]. Most experts recommend DP treatment with cranial orthoses, namely helmet-molding therapy, at 4 to 6 months of age [46,47,48,49]. However, a natural course of craniofacial symmetry from our study may provide a suggestion of observation only for those infants with mild plagiocephaly instead of helmet treatment. Furthermore, the effectiveness of helmet treatment in plagiocephaly patients could be compared to our craniofacial models of healthy infants. A study indicated a relationship between autism and a slightly larger head circumference, and a further application of 3D stereophotogrammetry may help obtain more accurate and relevant results [50]. For monitoring cranial growth and intracranial volume, CT and MRI series are convenient modalities, but they introduce infants to unnecessary radiation exposure and sedation management [51]. Although head circumference can be used as a surrogate indicator of intracranial volume [21], stereophotogrammetric images can provide extracranial volume, which provides a more precise and direct measurement of cranial growth.

## 5. Conclusions

In this study, the growth patterns of head height, depth, width, volume, and circumference; body height and weight; and BMI in infants up to 12 months of age were presented, and the normative craniofacial models at 1, 2, 4, 6, 9, and 12 months of age were established. Craniofacial asymmetry in infants that was gradually corrected by nature was discovered. Using these data, clinicians can evaluate infants with craniofacial deformities by comparing them with the values of healthy infants of the same age and sex.

## Figures and Tables

**Table 1:** Mean craniofacial norms and craniofacial asymmetry scores of male and female infants in each age group (*n* = 373).

	Head Height, mm (m ± SD)		Head Depth, mm (m ± SD)		Head Width, mm (m ± SD)		Head Volume, cm^3^ (m ± SD)	
Age/Sex	Male	Female	Male	Female	Male	Female	Male	Female
1 month	148.85 ± 7.38	146.28 ± 5.26	119.45 ± 7.09	119.55 ± 6.88	117.91 ± 5.34	115.46 ± 5.56	1061.10 ± 103.24	1027.38 ± 89.25
2 months	158.50 ± 6.18 *	152.41 ± 4.43	124.42 ± 6.15	122.35 ± 6.18	126.75 ± 6.58 *	122.66 ± 6.96	1267.04 ± 130.18 *	1186.42 ± 74.18
4 months	168.01 ± 6.44 *	162.32 ± 4.40	130.59 ± 5.97	128.97 ± 5.85	137.48 ± 6.02 *	129.57 ± 6.35	1545.80 ± 129.74 *	1425.21 ± 78.78
6 months	175.51 ± 5.49 *	170.11 ± 5.13	135.80 ± 6.17	134.66 ± 6.73	140.84 ± 6.70 *	134.06 ± 7.44	1748.97 ± 133.23 *	1634.00 ± 91.88
9 months	179.81 ± 5.59 *	175.07 ± 5.46	141.39 ± 6.52	140.40 ± 6.09	144.71 ± 7.64 *	138.80 ± 7.38	1920.66 ± 153.04 *	1809.08 ± 88.14
12 months	183.17 ± 5.45 *	178.67 ± 4.97	145.68 ± 6.97	145.49 ± 5.72	144.46 ± 8.08	141.41 ± 7.57	2031.78 ± 133.02 *	1935.86 ± 100.18
	**Body Height, cm (m ± SD)**		**Body Weight, kg (m ± SD)**		**BMI, kg/m^2^ (m ± SD)**		**Head Circumference, cm (m ± SD)**	
**Age/Sex**	**Male**	**Female**	**Male**	**Female**	**Male**	**Female**	**Male**	**Female**
1 month	53.30 ± 2.00	52.75 ± 2.03	4.46 ± 0.72	4.18 ± 0.53	15.62 ± 1.78	15.00 ± 1.37	37.09 ± 1.58	36.81 ± 0.98
2 months	57.60 ± 2.25 *	56.51 ± 1.73	5.79 ± 0.86 *	5.16 ± 0.53	17.38 ± 1.77 *	16.16 ± 1.36	39.05 ± 1.54	38.54 ± 1.04
4 months	62.88 ± 2.33 *	61.56 ± 2.26	7.31 ± 0.78 *	6.57 ± 0.67	18.48 ± 1.43 *	17.34 ± 1.67	41.39 ± 1.26 *	40.76 ± 1.02
6 months	67.10 ± 1.97 *	65.93 ± 2.37	8.41 ± 1.21 *	7.51 ± 0.77	18.63 ± 1.98 *	17.25 ± 1.39	43.24 ± 1.44 *	42.61 ± 0.91
9 months	70.88 ± 2.47	70.58 ± 2.67	9.19 ± 1.36	8.60 ± 1.02	18.27 ± 2.14 *	17.24 ± 1.61	44.95 ± 1.43 *	44.37 ± 0.80
12 months	74.71 ± 2.53	73.94 ± 2.68	9.71 ± 1.00	9.22 ± 1.14	17.39 ± 1.49	16.86 ± 1.83	45.99 ± 1.43	45.74 ± 0.86
**Craniofacial Asymmetry Score** **(mm ± SD)**								
**Age/Sex**			**Male**		**Female**		***p* Value**	
1 month			3.21 ± 2.04		2.87 ± 1.74		0.490	
2 months			2.90 ± 1.38		2.71 ± 1.45		0.586	
4 months			2.60 ± 1.36		2.31 ± 1.31		0.382	
6 months			2.52 ± 1.44		2.16 ± 1.03		0.265	
9 months			2.47 ± 1.26		1.91 ± 1.19		0.069	
12 months			2.13 ± 1.08		1.88 ± 0.87		0.326	

cm, centimeter; mm, millimeter; kg, kilogram; BMI, body mass index; m, mean; SD, standard deviation. * Statistical differences between male and female infants in the same selected month.

## Data Availability

Data can be provided upon the authors’ approval.

## Supplementary Materials

The following supporting information can be downloaded at: https://www.mdpi.com/article/10.3390/ijerph191912133/s1, Figure S1: Three-dimensional craniofacial photos of selected months in different sexes. (a) Three-dimensional craniofacial photos in selected months in a male infant (1, 2, 4, 6, 9, and 12 months). (b) Three-dimensional craniofacial photos in selected months in a female infant (1, 2, 4, 6, 9, and 12 months); Figure S2: Average 3D composite craniofacial meshes of selected months in different sexes. (a) Average 3D composite craniofacial meshes in selected months in a female infant (1, 2, 4, 6, 9, and 12 months). (b) Average 3D composite craniofacial meshes in selected months in a male infant (1, 2, 4, 6, 9, and 12 months).
