# Supplementary figures and images for: Craniofacial Growth and Asymmetry in Newborns: A Longitudinal 3D Assessment
Source: Int J Environ Res Public Health. 2022 Sep 25;19(19):12133. doi: 10.3390/ijerph191912133 (PMC9564900; doi:10.3390/ijerph191912133)

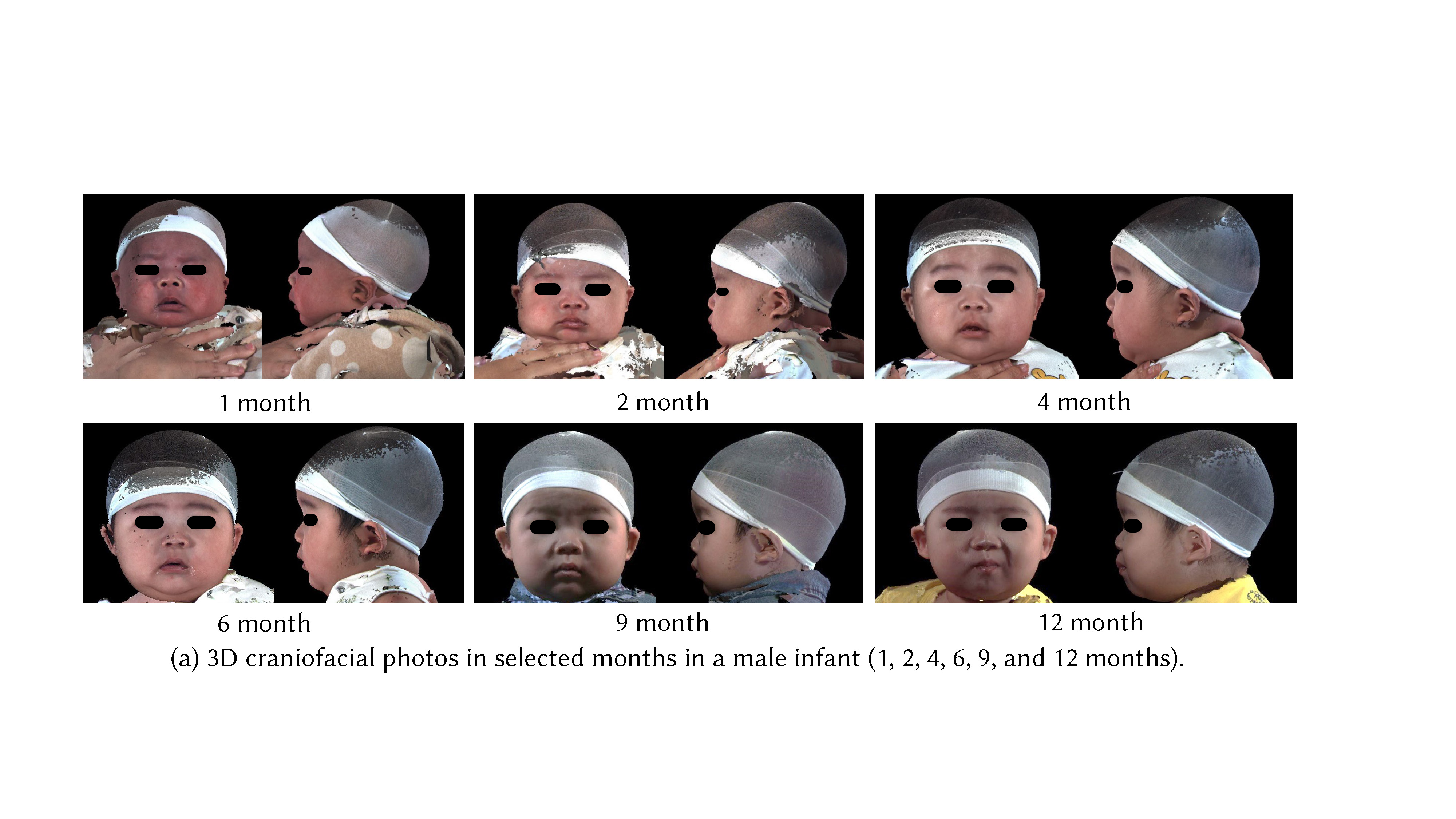

Supplement: Supplementary file 1 [file ijerph-19-12133-s001.zip › Supplements Figure S1a.png]

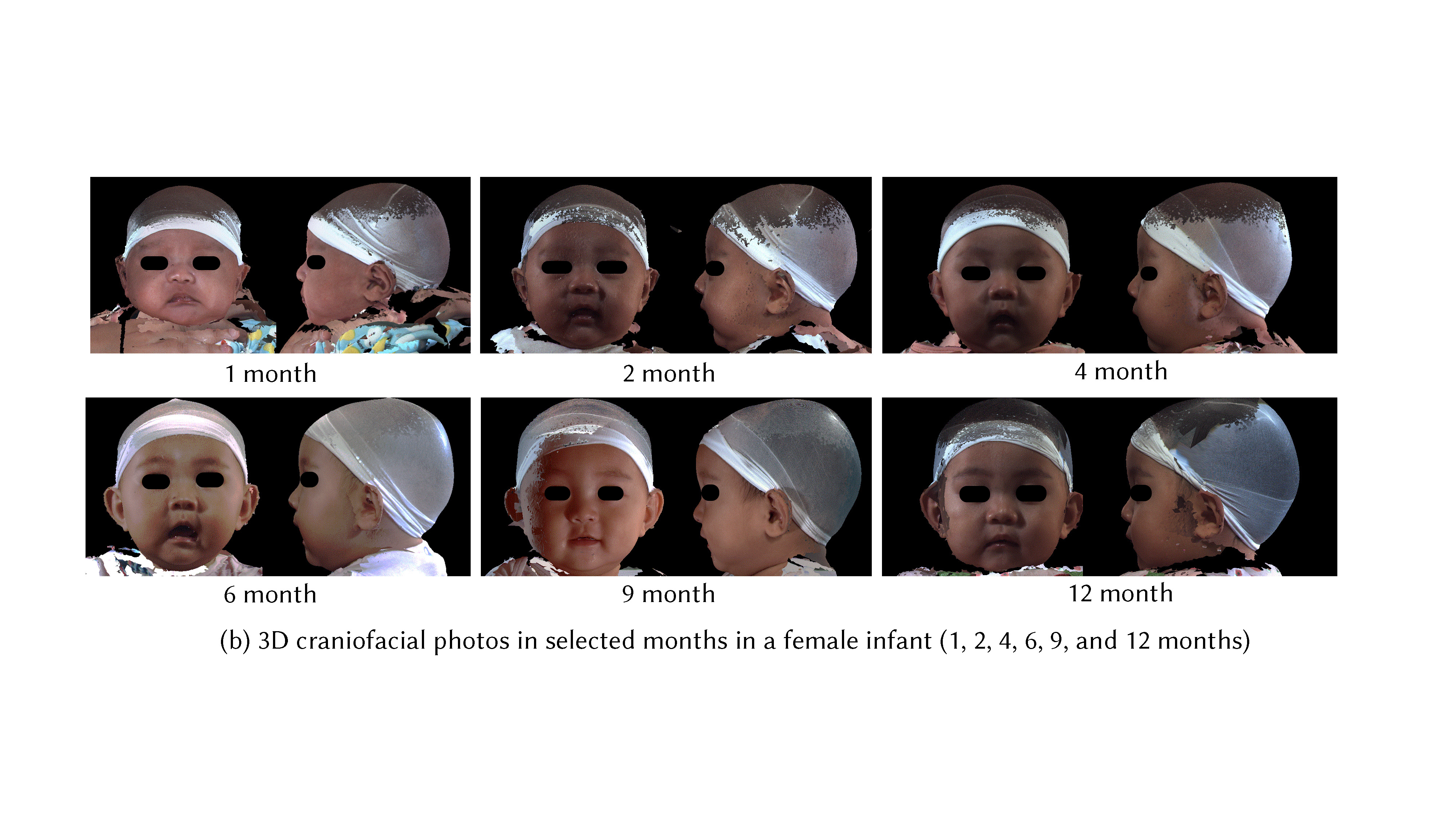

Supplement: Supplementary file 1 [file ijerph-19-12133-s001.zip › Supplements Figure S1b.png]

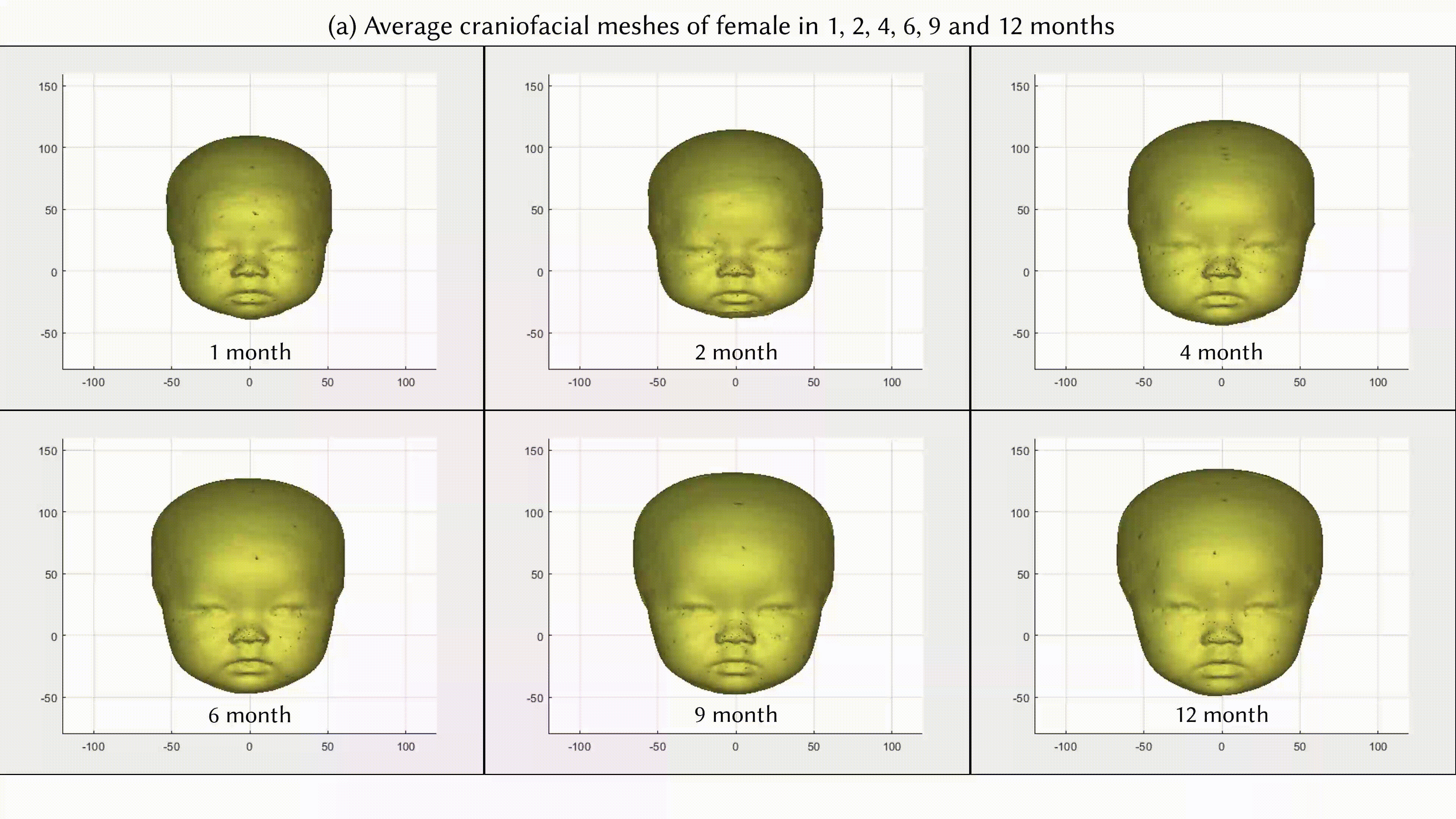

Supplement: Supplementary file 1 [file ijerph-19-12133-s001.zip › Supplements Figure S2a.gif]

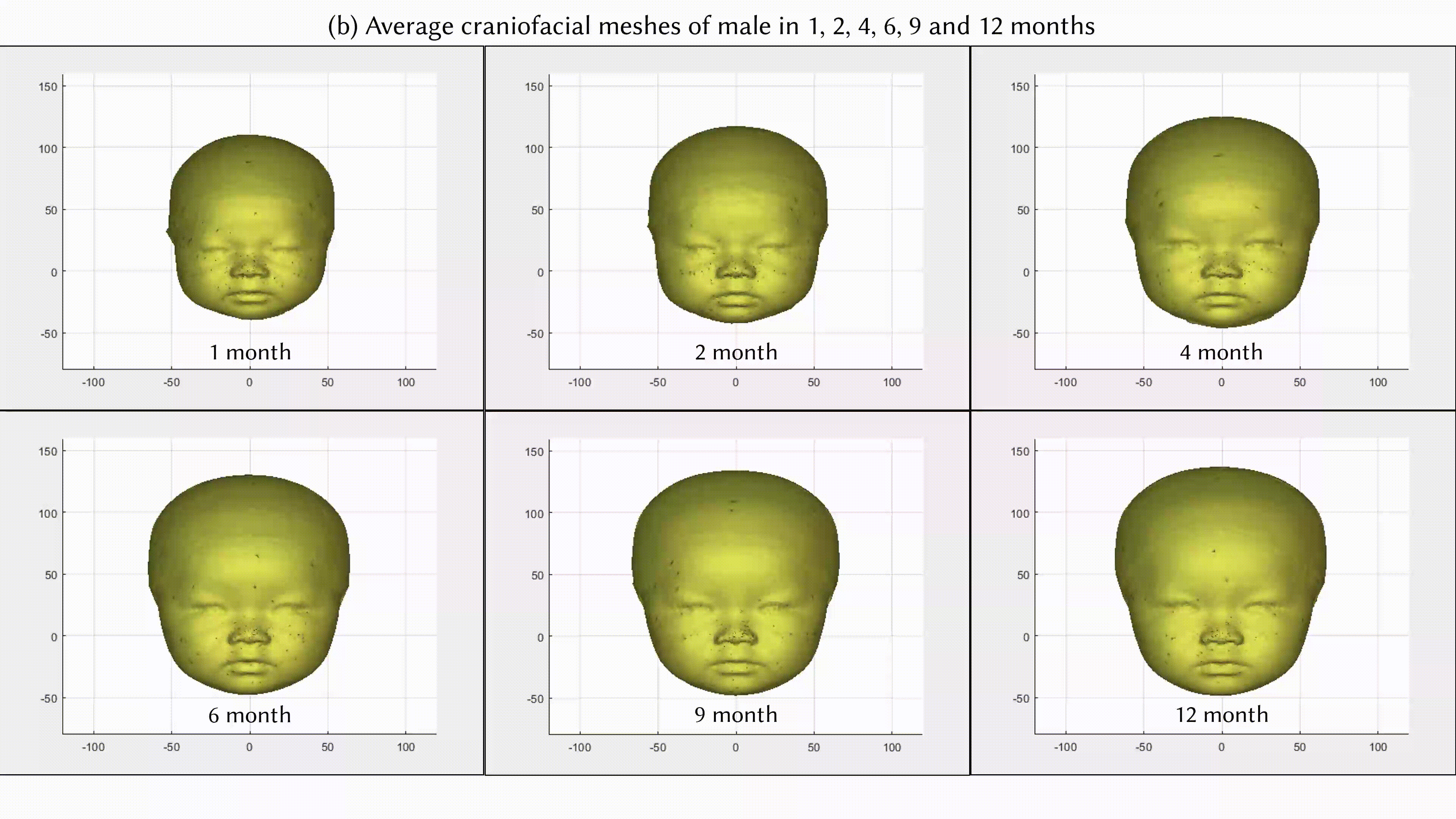

Supplement: Supplementary file 1 [file ijerph-19-12133-s001.zip › Supplements Figure S2b.gif]
